# Improvement in Phase Compatibility and Mechanical Properties of Poly(L-lactide)-*b*-poly(ethylene glycol)-*b*-poly(L-lactide)/thermoplastic Starch Blends with Citric Acid

**DOI:** 10.3390/polym15193966

**Published:** 2023-10-01

**Authors:** Prasong Srihanam, Yaowalak Srisuwan, Theeraphol Phromsopha, Apirada Manphae, Yodthong Baimark

**Affiliations:** 1 Biodegradable Polymers Research Unit, Department of Chemistry and Centre of Excellence for Innovation in Chemistry, Faculty of Science, Mahasarakham University, Mahasarakham 44150, Thailand; prasong.s@msu.ac.th (P.S.); yaowalak.s@msu.ac.th (Y.S.); theeraphol.p@msu.ac.th (T.P.); 2 Scientific Instrument Academic Service Unit, Faculty of Science, Mahasarakham University, Mahasarakham 44150, Thailand; apirada.m@msu.ac.th

**Keywords:** poly(lactic acid), poly(ethylene glycol), block copolymer, thermoplastic starch, citric acid

## Abstract

Flexible poly(L-lactide)-*b*-poly(ethylene glycol)-*b*-poly(L-lactide) block copolymer (PLLA-PEG-PLLA) bioplastic has been blended with low-cost thermoplastic starch (TPS) to prepare fully biodegradable bioplastics. However, the mechanical properties of PLLA-PEG-PLLA matrix decrease after the addition of TPS. In this work, citric acid (CA) was used as a compatibilizer to improve the phase compatibility and mechanical properties of PLLA-PEG-PLLA/TPS blends. TPS was first modified with CA (1.5 %wt, 3 %wt, and 4.5%wt) before melt blending with PLLA-PEG-PLLA. The PLLA-PEG-PLLA/modified TPS ratio was constant at 60/40 by weight. CA modification of TPS suppressed the crystallinity and enhanced the thermal stability of the PLLA-PEG-PLLA matrix, as determined through differential scanning calorimetry (DSC) and thermogravimetric analysis (TGA), respectively. The compatibility between the dispersed TPS and PLLA-PEG-PLLA phases was improved through modification of TPS with CA, as revealed by the smaller size of the co-continuous TPS phase from scanning electron microscopy (SEM) analysis. Increasing the hydrophilicity of the blends containing modified TPS confirmed the improvement in phase compatibility of the components. From the tensile test, the ultimate tensile strength, elongation at break, and Young’s modulus of the blends increased with the CA content. In conclusion, CA showed a promising behavior in improving the phase compatibility and mechanical properties of PLLA-PEG-PLLA/TPS blends. These PLLA-PEG-PLLA/modified TPS blends have potential to be used as flexible bioplastic products.

## 1. Introduction

Plastic waste is a serious environmental problem around the world that can be resolved by the use of biodegradable bioplastics instead of non-biodegradable petroleum-based plastics. For this purpose, poly(L-lactic acid) or poly(L-lactide) (PLLA) is an important biodegradable bioplastic for use in agricultural [1], packaging [2,3,4], sports [5], and biomedical [6] applications because of its non-toxicity, low cost, good mechanical strength, and easy processability [7,8,9]. However, the low flexibility, low toughness, slow crystallization rate, and slow biodegradation rate of PLLA limit its wide applications [9,10,11,12,13]. In our previous works [14,15,16], block copolymerization was used to prepare poly(L-lactide)-*b*-poly(ethylene glycol)-*b*-poly(L-lactide) (PLLA-PEG-PLLA) block copolymers, which showed a higher flexibility, higher toughness, faster crystallization rate, and faster biodegradation rate than the PLLA due to the flexibility and hydrophilicity of PEG middle-blocks.

PLLA has been widely blended with low-cost, bio-renewable, and biodegradable thermoplastic starch (TPS) to increase its competitive price [17,18] and to accelerate its biodegradation rate [16,17]. However, the poor compatibility between PLLA and TPS phases significantly decreased the mechanical properties of the PLLA matrices because of their low interfacial adhesion [17,18,19,20]. The PLLA-PEG-PLLA/TPS blends showed a better phase compatibility than that of the PLLA/TPS blends [16,20]. The dispersed TPS represented a co-continuous phase in the PLLA-PEG-PLLA matrix. This is due to the enhanced hydrophilic PEG middle-blocks’ phase compatibility between the PLLA end-blocks and TPS. However, tensile properties of the PLLA-PEG-PLLA matrix decreased as the TPS content increased.

It has been reported that the phase compatibility of the PLLA/TPS blends has been improved by using compatibilizers such as maleic anhydride [21,22], formamide [23], and citric acid (CA) [24]. Among these compatibilizers, CA is a low-cost and non-toxic compatibilizer. The TPS modified with CA exhibited good phase compatibility with PLLA matrix [24] and polyethylene/natural rubber matrix [25], improving the tensile properties including tensile stress and strain at break of the polymer matrices. To the best of our knowledge, PLLA-PEG-PLLA/CA-modified TPS blends have not been reported so far. We hypothesized that the phase compatibility and mechanical properties of the PLLA-PEG-PLLA/TPS blends could be improved through modification of TPS with CA.

The objective of this paper was to investigate the effect of CA modification on the thermal properties, phase morphology, and tensile properties of the PLLA-PEG-PLLA/TPS blends. For this purpose, the TPS was first modified with 1.5 %wt, 3 %wt, and 4.5 %wt CA before melt-blending with PLLA-PEG-PLLA. The ratio of PLLA-PEG-PLLA/modified TPS was constant at 60/40 (*w*/*w*). The 60/40 (*w*/*w*) PLLA-PEG-PLLA/unmodified TPS blend was also prepared using the same method for comparison. These bio-based PLLA-PEG-PLLA/TPS blends with a more flexible and faster biodegradation rate than the PLLA/TPS blends could be used as single-use bioplastics [16]. Increasing the mechanical properties of the PLLA-PEG-PLLA/TPS blends should ensure that they are more widely used. Therefore, these blends have broad and sustainable application prospects in the agricultural and packaging fields.

## 2. Materials and Methods

### 2.1. Materials

A chain-extended PLLA-PEG-PLLA was synthesized through ring-opening polymerization of L-lactide monomer in the presence of a Joncryl ADR4368 chain extender (2 parts per hundred of resin) under nitrogen atmosphere at 165 °C for 6 h, as described in our previous works [15,26]. The number-averaged molecular weight (*M_n_*) and dispersity (*Ð*) of the PLLA-PEG-PLLA obtained from gel permeation chromatography (GPC) were 108,500 and 2.2, respectively [26]. Thermoplastic starch (TPS, TapioplastTM) with 79% amylopectin and citric acid (CA) were supplied by SMS Corporation Co., Ltd. (Pathum Thani, Thailand) and Merck (Rahway, NJ, USA), respectively. This TPS was prepared using 20 %wt glycerol as a plasticizer.

### 2.2. Modification of TPS with CA

TPS was modified with CA through melt blending at 150 °C for 10 min using an internal mixer (HAAKE Polylab OS Rheomix batch-mixer, Thermo Scientific, Waltham, MA, USA) with a 100 rpm rotor speed. The TPS and CA were dried in a vacuum oven at 50 °C overnight before use. The CA contents of 1.5 %wt, 3 %wt, and 4.5 %wt were investigated. The unmodified TPS was also prepared using the same method for comparison.

### 2.3. Preparation of PLLA-PEG-PLLA/TPS Blends

PLLA-PEG-PLLA/modified TPS blends were prepared through melt blending at 180 °C for 5 min using an internal mixer with a 100 rpm rotor speed. The PLLA-PEG-PLLA and modified TPS were dried in a vacuum oven at 50 °C overnight before use. The PLLA-PEG-PLLA/modified TPS weight ratio was kept constant at 60/40. 

Blend films were prepared through compression molding using a hot-press machine (Auto CH Carver). The blend samples were heated at 180 °C without compression force for 3 min before hot pressing at 180 °C under 5 MPa load for 1 min. The blend films were then cooled to room temperature using a water-cooled press under 5 MPa load for 1 min. The blends were dried in a vacuum oven at 50 °C overnight before use. The obtained film thicknesses were around 0.2 mm. The 60/40 (*w*/*w*) PLLA-PEG-PLLA/unmodified TPS blend sample was also prepared using the same method for comparison.

### 2.4. Characterization of Modified TPS and PLLA-PEG-PLLA/TPS Blends

The FTIR spectra of the samples were determined using a Fourier-transform infrared (FTIR) spectrometer (Invenio-S, Bruker, Germany) in an attenuated total reflectance (ATR) accessory over the wavenumber range of 500–4000 cm^−1^. A resolution of 4 cm^−1^ and accumulated scans of 32 were used. 

The thermal transition properties of the samples were measured using a differential scanning calorimeter (DSC, Pyris Diamond, PerkinElmer, Waltham, MA, USA) over the temperature range of 0–200 °C. For DSC heating scan, the samples were held at 200 °C for 3 min to remove thermal history followed by fast quenching to 0 °C before scan using a heating rate of 10 °C/min under a nitrogen gas flow. The degree of crystallinity (*Xc*) of the samples was calculated using Equation (1).

*X_c_* (%) = [(Δ*H_m_* − Δ*H_cc_*)/(93.6 × *W_PLLA_*)] × 100 (1)

where ∆*H_m_* and ∆*H_cc_* are enthalpies of melting and cold crystallization, respectively. The Δ*H_m_* of 100%*X_c_* PLLA is 93.6 J/g [16]. *W_PLLA_* is the PLLA weight fraction in the blends.

For DSC cooling scan, the samples were held at 200 °C for 3 min to erase thermal history before scanning from 200 to 0 °C using a cooling rate of 10 °C/min under a nitrogen gas flow.

The thermal decomposition properties of the samples were carried out using a thermogravimetric analyzer (TGA, SDT Q600, TA-Instrument, New Castle, DE, USA) in a temperature range of 50–1000 °C using a heating rate of 20 °C/min under a nitrogen gas flow.

The film morphology of cryogenically fractured surfaces of the samples were observed using a scanning electron microscope (SEM, JSM-6460LV, JEOL, Tokyo, Japan). The samples were sputter-coated with gold before scan. In addition, the film morphology of TPS-etched surfaces was also observed by immersing the cryogenically fractured films in aqueous HCl solution (6 N) for 3 h to remove the TPS followed by washing and drying in a vacuum oven at room temperature overnight before SEM analysis [20].

The water contact angle of the film samples was measured using a contact angle goniometer (DataPhysics, OCA 11, Filderstadt, Germany). The water contact angle was recorded after a 2.5 μL water droplet was dropped onto the film surface for 15 s. The averaged result was an average value of five measurements.

The tensile properties of the film samples (100 mm × 10 mm) were tested using a universal mechanical tester (LY-1066B, Dongguan Liyi Environmental Technology Co., Ltd., Dongguan, China) at 25 °C with a crosshead speed of 50 mm/min, a gauge length of 50 mm, and a load cell of 100 kg. At least five determinations were tested for each sample.

## 3. Results and Discussion

The TPS was first modified with CA through melt processing. Ester bonds of the modified TPS were formed through esterification reaction between hydroxyl groups of TPS and carboxylic acid groups of CA [24]. Meanwhile, the molecular weight and viscosity of starch decreased through CA-catalyzed hydrolysis [27,28]. The CA-modified TPS was further melt-blended with PLLA-PEG-PLLA. The interfacial adhesion and phase compatibility of PLLA-PEG-PLLA/TPS blends were very sensitive to changes in their thermal and mechanical properties and were therefore of practical and fundamental interest in this work.

### 3.1. FTIR Spectroscopy of the Unmodified and Modified TPS

**Figure 1:**
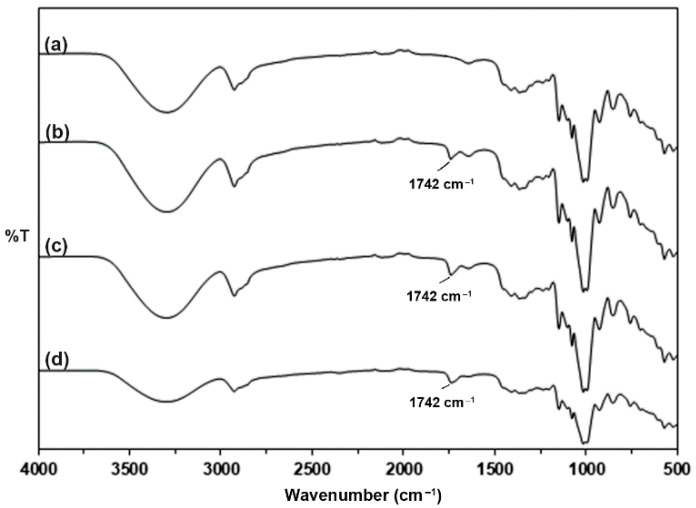
ATR-FTIR spectra of (**a**) unmodified TPS and modified TPS with CA contents of (**b**) 1.5 %wt, (**c**) 3 %wt, and (**d**) 4.5 %wt.

Chemical structures of the unmodified and modified TPS were studied from ATR-FTIR spectra as presented in Figure 1. The unmodified TPS in Figure 1a showed peaks at 3300 cm^−1^, 2926 cm^−1^, and 1635 cm^−1^, which refer to the stretching of hydroxyl groups, stretching of C-H groups, and bending of hydroxyl groups of starch, respectively [20,24,29]. For the modified TPS, a new peak at 1742 cm^−1^ related to ester carbonyl groups appeared and the peak intensity at 3300 cm^−1^ of hydroxyl groups of TPS significantly decreased as the CA content increased, suggesting that the esterification process between hydroxyl groups of TPS and carboxylic acid groups of CA had occurred to form ester bonds in modified TPS [24,30,31,32]. In addition, the vibration of carbonyl groups of acids for pure CA exhibited a peak at 1720 cm^−1^ [33]. This peak was not detected in modified TPS, supporting the conclusion that an esterification reaction between TPS and CA had occurred [33]. Thus, the FTIR results confirmed that the linkages between TPS and CA were formed in the modified TPS.

### 3.2. FTIR Spectroscopy of the PLLA-PEG-PLLA/TPS Blends

**Figure 2:**
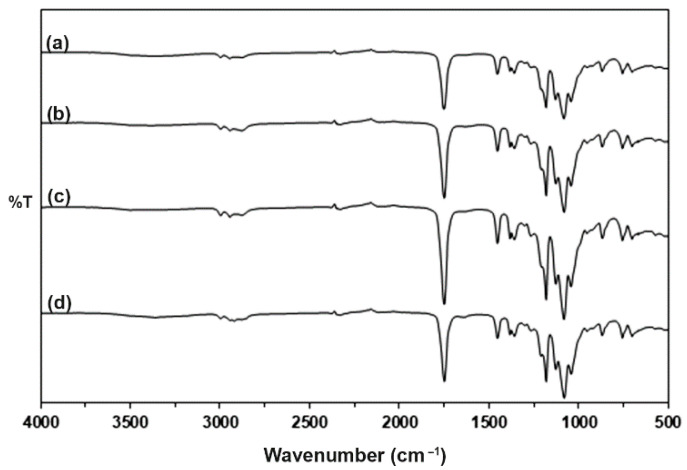
ATR-FTIR spectra of (**a**) PLLA-PEG-PLLA/unmodified TPS blend film and PLLA-PEG-PLLA/modified TPS blend films with CA contents of (**b**) 1.5 %wt, (**c**) 3 %wt, and (**d**) 4.5 %wt.

Figure 2 shows ATR-FTIR spectra of the PLLA-PEG-PLLA/TPS blend films to investigate their chemical functional groups. The peaks at 3300 cm^−1^ due to the stretching of hydroxyl groups of TPS decreased in intensity because it was blended with 60 %wt PLLA-PEG-PLLA containing a lower content of hydroxyl groups [20]. The ATR-FTIR spectrum of the PLLA-PEG-PLLA/unmodified TPS blend in Figure 2a showed a peak at 1752 cm^−1^, attributed to the stretching of ester carbonyl groups for the PLLA end-blocks; peaks at 2995 cm^−1^, 2945 cm^−1^, and 2878 cm^−1^, attributed to the stretching of C-H groups for the PLLA end-blocks; peaks at 2945 cm^−1^ and 2878 cm^−1^, attributed to the stretching of asymmetric and symmetric of –CH_2_ groups for the PEG middle-blocks, respectively; as well as a peak at 2920 cm^−1^, attributed to the stretching of C-H groups for the TPS [20,29,34]. The ester carbonyl peaks had slightly shifted to lower wavenumbers at 1751 cm^−1^, 1751 cm^−1^, and 1750 cm^−1^ for the PLLA-PEG-PLLA/modified TPS blends with CA contents of 1.5 %wt, 3 %wt, and 4.5 %wt, respectively, suggesting that the CA modification of TPS enhanced interactions between carbonyl groups of PLLA end-blocks and hydroxyl groups of TPS.

### 3.3. Thermal Transition Properties of the PLLA-PEG-PLLA/TPS Blends

**Figure 3:**
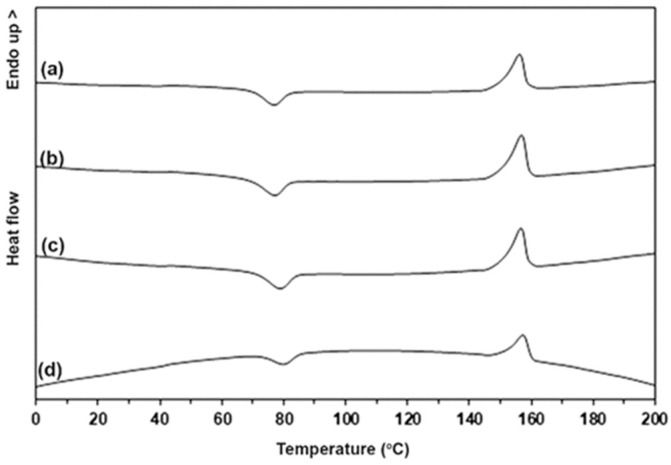
DSC heating curves of (**a**) PLLA-PEG-PLLA/unmodified TPS blend and PLLA-PEG-PLLA/modified TPS blends with CA contents of (**b**) 1.5 %wt, (**c**) 3 %wt, and (**d**) 4.5 %wt.

The thermal transition properties including the glass transition temperature (*T_g_*), cold crystallization temperature (*T_cc_*), melting temperature (*T_m_*), and degree of crystallinity (*X_c_*) of the blends were determined from DSC heating curves as shown in Figure 3, and Table 1 summarizes the DSC results. The *T_g_* and *T_m_* values of the blends containing unmodified TPS were 42 °C and 159 °C, respectively. All the blends containing modified TPS had *T_g_* and *T_m_* values near to those of the blends containing unmodified TPS. Therefore, the CA modification of TPS did not affect the *T_g_* values of the PLLA-PEG-PLLA matrix. The *T_m_* peaks of the blends containing modified TPS slightly shifted to lower temperatures, suggesting that the blending of modified TPS induced imperfect PLLA crystallites in the blends. The *T_cc_* peaks of the blends slightly shifted to higher temperatures and the *X_c_* values decreased as the CA content increased. The shifting to higher temperature of the *T_cc_* peak of the PLLA-based blends implied that the crystallization of PLLA upon DSC heating scan was suppressed [12,20] and then the *X_c_* of the PLLA decreased. Thus, it can be concluded from the results that the CA modification of TPS decreased the crystallizability of PLLA end-blocks in the blends.

**Figure 4:**
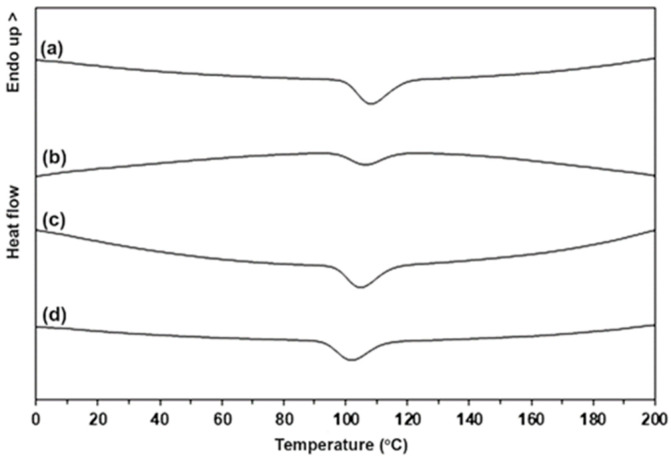
DSC cooling curves of (**a**) PLLA-PEG-PLLA/unmodified TPS blend and PLLA-PEG-PLLA/modified TPS blends with CA contents of (**b**) 1.5 %wt, (**c**) 3 %wt, and (**d**) 4.5 %wt.

The crystallization property of the blends was also investigated from DSC cooling curves as presented in Figure 4. The exothermic peak in Figure 4 was assigned to crystallization temperature (*T_c_*). The *T_c_* peak of the blend containing unmodified TPS was at 108 °C. The blends containing modified TPS were 106 °C, 105 °C, and 102 °C for the CA contents of 1.5 %wt, 3 %wt, and 4.5 %wt, respectively. The *T_c_* peaks of the blends shifted to lower temperatures with the increase in CA content. The shifting to lower temperature of the *T_c_* peak suggested that the PLLA crystallization upon DSC cooling scan was inhibited [20,26]. This supports that the crystallized ability of PLLA end-blocks in the blends was suppressed when the TPS was modified with CA and the CA content increased. These results may be due to the fact that acidic hydrolysis of TPS with the CA modification decreased the molar mass of TPS [27,28,30] to enhance the phase compatibility of the blend components and to inhibit the crystallization of PLLA end-blocks in the blends [20]. The phase compatibility of the blends was observed using SEM analysis, which is described later.

### 3.4. Thermal Decomposition Properties of the PLLA-PEG-PLLA/TPS Blends

**Figure 5:**
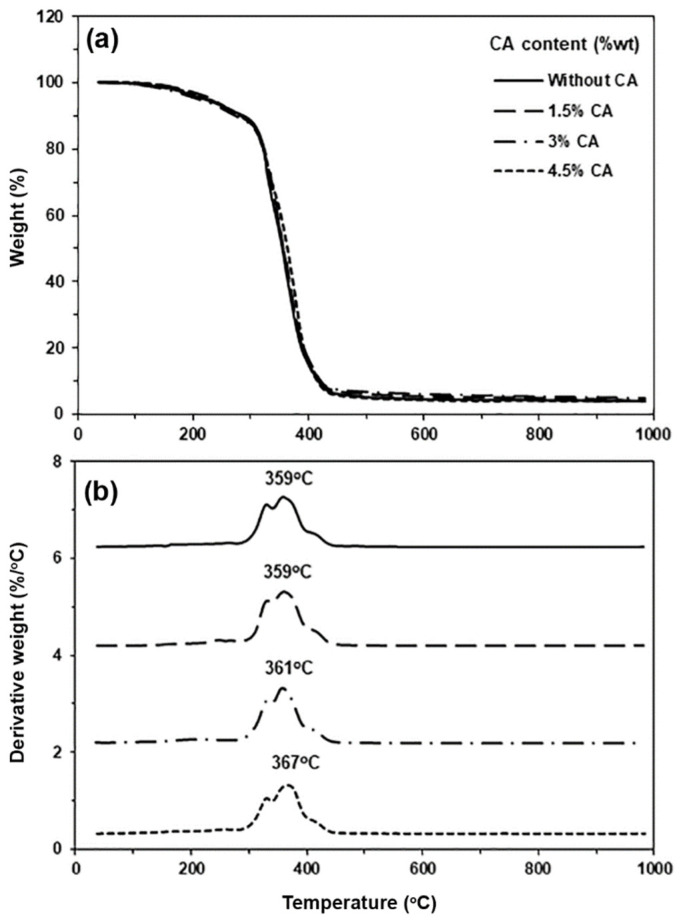
Thermograms of (**a**) TG and (**b**) DTG for PLLA-PEG-PLLA/TPS blends with various CA contents.

The thermal decomposition properties of the blends were studied from thermogravimetric (TG) and derivative TG (DTG) thermograms, as shown in Figure 5, and the results are summarized in Table 2. The TG thermograms of the blends exhibited two weight-loss steps in the ranges of 100–350 °C and 350–450 °C due to thermal decompositions of TPS and PLLA-PEG-PLLA components, respectively [16,20,32]. From Table 2, the decomposition temperature at 50% weight loss (*50%-T_d_*) of the PLLA-PEG-PLLA/unmodified TPS blend was 353 °C. The *50%-T_d_* values of the blends shifted to higher temperatures with increasing CA content. The weight remaining at 1000 °C of all the blends attributed to ash of TPS phases was near the range of 3.86–4.22%.

The temperatures at the maximum decomposition rate for PLLA end-blocks (*PLLA-T_d,max_*) and for PEG middle-blocks (*PEG-T_d,max_*) were 359 °C and 416 °C, respectively, as also reported in Table 2. It was found that the *PLLA-T_d,max_* peaks shifted to higher temperatures when the PLLA-PEG-PLLA was blended with modified TPS. However, *PEG-T_d,max_* peaks did not significantly shift after the addition of both the unmodified and modified TPS. It should be noted that pyrolysis of the starch component revealed shoulder *T_d,max_* peaks at 330 °C [20,35]. From the TG and DTG results, it can be concluded that CA modification of TPS is able to improve the thermal stability of the PLLA end-block in the blends. It has been reported that the products of starch thermal decomposition can improve the thermal stability of the polymer matrix in the compatible blends by protecting the polymer matrix during heat degradation [20,35,36]. Thus, better phase-compatible PLLA-PEG-PLLA/modified TPS blends exhibited a higher thermal stability of the PLLA end-blocks. The TGA results also suggested that the compatibility between PLLA-PEG-PLLA and TPS phases was improved through CA modification of TPS and by increasing CA content, which is confirmed later using SEM analysis.

### 3.5. Phase Compatibility of the PLLA-PEG-PLLA/TPS Blends

**Figure 6:**
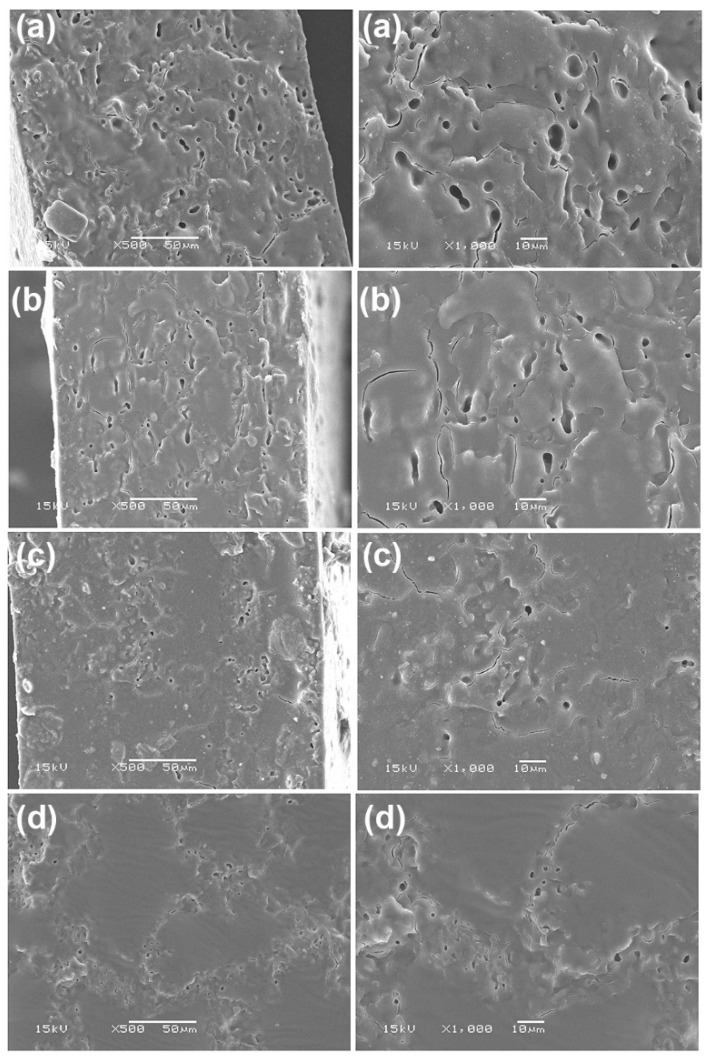
SEM images of cryogenically fractured surfaces for (**a**) PLLA-PEG-PLLA/unmodified TPS blend film and PLLA-PEG-PLLA/modified TPS blend films with CA contents of (**b**) 1.5 %wt, (**c**) 3 %wt, and (**d**) 4.5 %wt by magnifications of (**left** column) ×500 and (**right** column) ×1000 (bar scales = 50 µm and 10 µm for left and right columns, respectively).

SEM images of cryogenically fractured surfaces of the blend films were used to investigate the effect of CA on the compatibility between PLLA-PEG-PLLA and TPS phases, as shown in Figure 6. The TPS existed as a dispersed phase in the PLLA-PEG-PLLA matrix. Empty voids in the film samples were observed that resulted from detachment of the TPS phases during the cryo-fractured step. This was attributed to phase separation between PLLA-PEG-PLLA and TPS, suggesting that the interfacial adhesion between the blend components was relatively weak [20,34,36]. It is clearly seen that the empty voids in the PLLA-PEG-PLLA/modified TPS blends were minimized. The size and amount of TPS phases decreased significantly with the increase in CA content, implying that the phase compatibility in the blends was improved [24,25,27,34]. Thus, CA modification of TPS enhanced the interfacial adhesion between the blended components.

**Figure 7:**
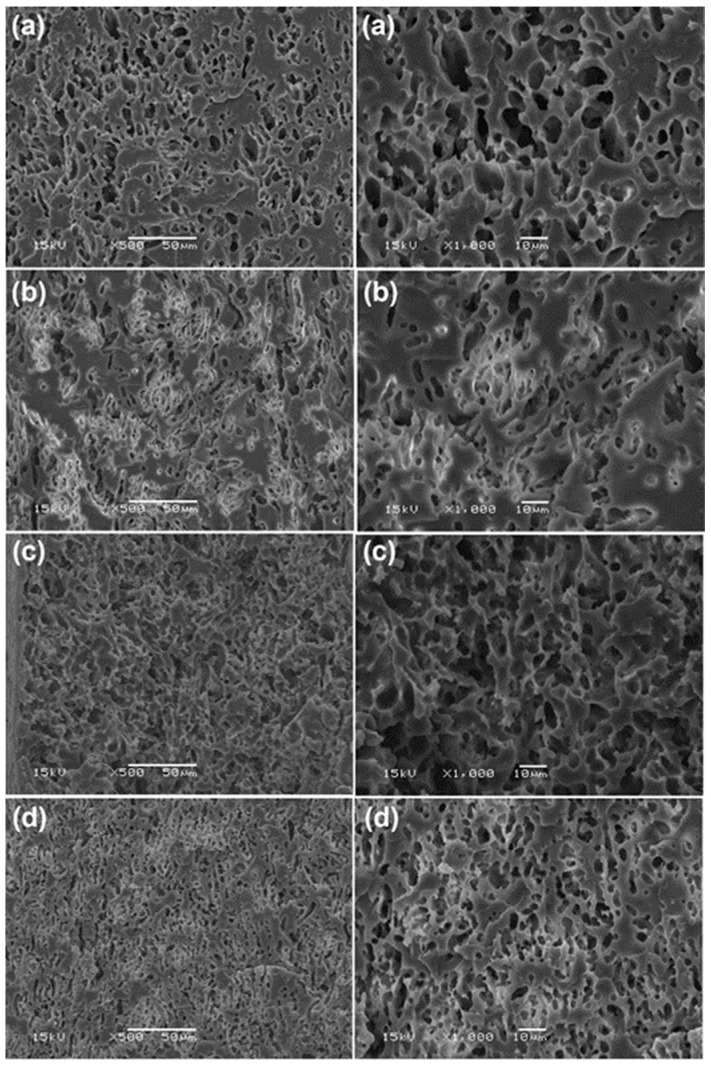
SEM images of cryogenically fractured surfaces after TPS extraction with HCl solution for (**a**) PLLA-PEG-PLLA/unmodified TPS blend film and PLLA-PEG-PLLA/modified TPS blend films with CA contents of (**b**) 1.5 %wt, (**c**) 3 %wt, and (**d**) 4.5 %wt by magnifications of (**left** column) ×500 and (**right** column) ×1000 (bar scales = 50 µm and 10 µm for left and right columns, respectively).

The phase compatibility of the blends was also investigated from their cryogenically fractured surfaces after etching the TPS phases, as shown in Figure 7. The porous structures of the fracture surfaces were observed, indicating that the dispersed TPS was a co-continuous phase in the PLLA-PEG-PLLA matrix. The PEG middle-blocks acted as compatibilization sites to improve interfacial adhesion between the PLLA end-blocks and TPS because of the good miscibility between PLLA and PEG [37] as well as between starch and PEG [38,39].

From Figure 7, it was found that the co-continuous TPS phases in the blends became finer-dispersion and smaller-size when the TPS was modified with CA and the CA content was increased. The results suggested that the CA modification of TPS improved the compatibility between the PLLA-PEG-PLLA and TPS phases [27,34]. The TPS has been modified with CA to improve the phase compatibility with the PLLA [24], HDPE/NR [25], and PE [40] matrices. This may be explained by the fact that the molecular weight and viscosity of TPS decreased through the acidolysis process with CA to enhance TPS dispersion in the blend matrix during melt blending [27,28]. In addition, the entanglement of starch chains was reduced by the acidity of CA to increase the fluidity of the TPS [41].

### 3.6. Water Contact Angles of the PLLA-PEG-PLLA/TPS Blends

**Figure 8:**
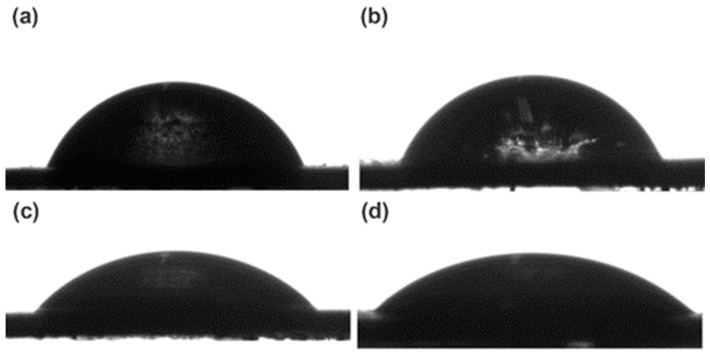
Water contact angles of (**a**) PLLA-PEG-PLLA/unmodified TPS blend film and PLLA-PEG-PLLA/modified TPS blend films with CA contents of (**b**) 1.5 %wt, (**c**) 3 %wt, and (**d**) 4.5 %wt.

Water contact angle measurement was used to investigate the hydrophilicity of the film samples as shown in Figure 8 and summarized in Table 3. The water contact angle of 60/40 (*w*/*w*) PLLA-PEG-PLLA/unmodified TPS film was 55.3°, consistent with the result of our previous work [20]. The addition of hydrophilic TPS decreased the water contact angle of the pure PLLA-PEG-PLLA film (69.1°). As the modified TPS was blended and the CA content was increased, the water contact angle gradually decreased, indicating that the hydrophilicity of the blends increased with the CA content, indicating that the TPS richness on film surface was increased. The notable reduction in water contact angle of the blend films after CA modification of TPS can be attributed to increasing the dispersion of TPS on the PLLA-PEG-PLLA matrix according to the above SEM analysis.

### 3.7. Mechanical Properties of the PLLA-PEG-PLLA/TPS Blends

**Figure 9:**
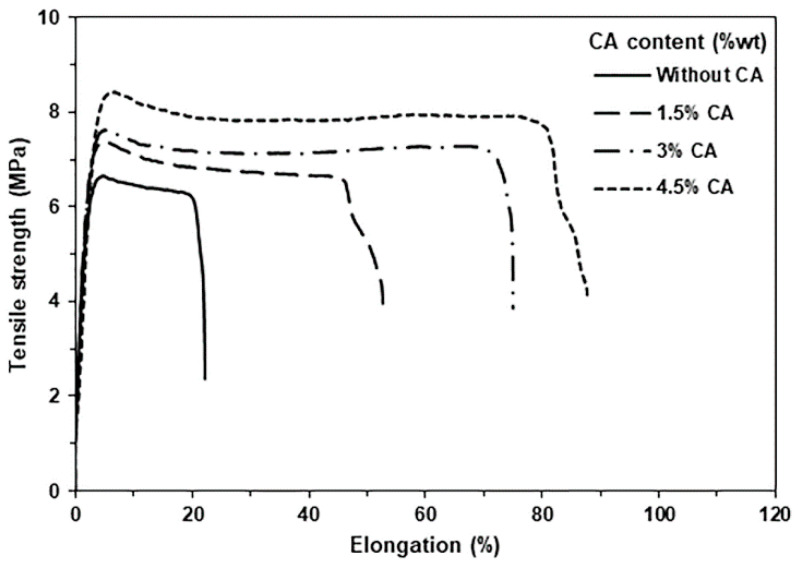
Tensile curves of PLLA-PEG-PLLA/TPS blend films with various CA contents.

The tensile test was used to determine the mechanical properties of the film samples, as illustrated in Figure 9, and the tensile properties are also reported in Table 3. The values of ultimate tensile strength, elongation at break, and Young’s modulus of the PLLA-PEG-PLLA/unmodified TPS blend film were 6.1 MPa, 24%, and 84 MPa, respectively. These tensile properties of the blend films increased up to 7.4 MPa, 51%, and 89 MPa for the ultimate tensile strength, elongation at break, and Young’s modulus, respectively, when the TPS was modified with 1.5 %wt CA before blending. The tensile properties of the blends also increased as the CA content increased. The tensile results indicated that CA modification of TPS improved the tensile properties of the blend films.

It has been reported that the CA modification of TPS improved the tensile properties of the PLLA/TPS [24], HDPE/NR/TPS [25], and PE/TPS [40] blends. The improvement in the tensile properties occurred from the finer dispersion and smaller size of the TPS phase in the blend matrix [25]. Good dispersion and compatibility of the TPS phase in the blend matrix enhanced the mechanical properties of blends by improving the bond strength of the interfacial adhesion between blend components [24,42].

## 4. Conclusions

In summary, TPS modified with different CA contents (1.5 %wt, 3 %wt, and 4.5%wt) was prepared before melt blending with PLLA-PEG-PLLA to investigate their effect on the thermal properties, phase compatibility, and mechanical properties of PLLA-PEG-PLLA/TPS blends. The TPS could react with CA to form modified TPS as studied through FTIR analysis. The crystallized ability of the PLLA-PEG-PLLA matrices was suppressed and the thermal stability of PLLA-PEG-PLLA matrices was improved through the CA modification of TPS as determined through DSC and TGA analyses, respectively. The results of SEM and water contact angle measurement showed that the blends with higher CA contents exhibited better phase compatibility and higher hydrophilicity, respectively. The mechanical properties of the blends revealed through the tensile test indicated that the tensile properties including ultimate tensile strength, elongation at break, and Young’s modulus of the blends increased as the CA content increased. It can be concluded that CA acted as an excellent compatibilizer for PLLA-PEG-PLLA/TPS blends through the improvement in their phase compatibility and mechanical properties. These PLLA-PEG-PLLA/modified TPS blends have the potential to be developed further for use as fully bio-renewable and biodegradable materials for agricultural and packaging applications.

## Figures and Tables

**Table 1:** Thermal transition properties of PLLA-PEG-PLLA/TPS blends.

CA Content (%wt)	*T_g_* (°C)	*T_cc_* (°C)	*T_m_* (°C)	*X_c_* (%)
- 1.5 3 4.5	42 42 42 42	77 77 79 80	159 157 158 157	19.5 19.7 16.5 16.1

**Table 2:** Thermal decomposition properties of PLLA-PEG-PLLA/TPS blends.

CA Content (%wt)	*50%-T_d_* (°C) ^a^	Residue Weight at 1000 °C (%) ^a^	*PLLA-T_d,max_* (°C) ^b^	*PEG-T_d,max_* (°C) ^b^
- 1.5 3 4.5	353 356 357 364	3.98 3.86 4.22 3.94	359 359 361 367	416 414 415 416

^a^ Obtained from TG thermograms. ^b^ Obtained from DTG thermograms.

**Table 3:** Water contact angles and tensile properties of PLLA-PEG-PLLA/TPS blend films.

CA Content (%wt)	Water Contact Angle (°)	Ultimate Tensile Strength (MPa)	Elongation at Break (%)	Young’s Modulus (MPa)
- 1.5 3 4.5	55.3 ± 4.5 51.1 ± 6.3 41.9 ± 4.2 35.5 ± 5.1	6.1 ± 0.7 7.4 ± 0.5 7.5 ± 1.2 8.3 ± 1.1	24 ± 6 51 ± 5 73 ± 7 80 ± 12	84 ± 15 89 ± 12 103 ± 14 107 ± 21

## Data Availability

Not applicable.
